# Evaluating ChatGPT’s Efficacy in Pediatric Pneumonia Detection From Chest X-Rays: Comparative Analysis of Specialized AI Models

**DOI:** 10.2196/67621

**Published:** 2025-01-10

**Authors:** Nitin Chetla, Mihir Tandon, Joseph Chang, Kunal Sukhija, Romil Patel, Ramon Sanchez

**Affiliations:** 1 Department of Radiology University of Virginia School of Medicine Charlottesville, VA United States; 2 Department of Orthopaedics Albany Medical College Albany, NY United States; 3 Department of Radiology University of Passau Passau Germany; 4 Department of Emergency Medicine Kaweah Health Medical Center Visalia, CA United States; 5 Department of Radiology Children's National Hospital Washington, DC United States

**Keywords:** artificial intelligence, ChatGPT, pneumonia, chest x-ray, pediatric, radiology, large language models, machine learning, pneumonia detection, diagnosis, pediatric pneumonia

## Introduction

Recent studies have demonstrated the versatility of ChatGPT in health care [1]. In contrast, convolutional neural networks (CNNs) have an established history in medical imaging, particularly in identifying pneumonia from chest x-rays. CNNs are a class of deep learning algorithms that recognize patterns in images, making them invaluable tools in radiology and other imaging-based diagnostics [2]. Numerous studies demonstrate CNNs’ effectiveness in medical imaging [3].

With advancements and developments in artificial intelligence (AI) technology, this research aims to evaluate the effectiveness of using ChatGPT-4 to detect pneumonia on x-ray images and compare its performance with specialized CNNs. These technologies could address radiologist shortages.

Community-acquired pneumonia incidence has reached 450 million cases worldwide annually [4]. In diagnosing pneumonia, a clinical history, physical examination, and laboratory tests are required, but clinical guidelines consider chest x-ray as the gold standard for distinguishing pneumonia from other respiratory tract infections [5]. However, interobserver agreement has been poor in chest radiographs of pediatric pneumonia [6]. Technological improvements such as ChatGPT and AI can help detect and diagnose pediatric pneumonia.

## Methods

This study used a dataset of chest x-rays from the Kaggle dataset “Chest X-Ray Images (Pneumonia),” originally sourced from the Guangzhou Women and Children’s Medical Center [3,7]. The dataset consists of 5863 pneumonia and normal chest x-ray images. The images were selected from retrospective cohorts of pediatric patients, aged 1-5 years, who underwent anterior-posterior chest x-rays as part of their workup. For quality assurance, the diagnoses associated with the images were graded by three expert physicians. The dataset includes bacterial and viral pneumonia cases but does not specify the type of pneumonia or distinguish between simple and complicated pneumonia.

The study used a subset of this dataset, consisting of 500 x-rays with pneumonia and 500 without pneumonia. Each image is stored in a subfolder labeled “Pneumonia” or “Normal,” enabling straightforward categorization and access. ChatGPT-4 was then prompted with “Based on the image, does the patient have A) pneumonia or B) no pneumonia? Only output the answer as A or B.” The results were analyzed.

## Results

**Figure 1:**
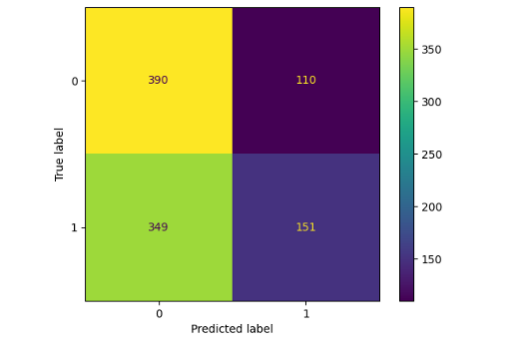
Confusion matrix of ChatGPT-4 Turbo.

ChatGPT-4 Turbo was biased toward the answer nonpneumonia (Table 1 and Figure 1). The substantial bias affects the statistical measures used. ChatGPT-4o performs slightly better overall, except in sensitivity and specificity.

**Table 1:** Statistical overview table of results of ChatGPT-4 Turbo and GPT-4o.

Statistic	ChatGPT-4 Turbo	ChatGPT-4o
Accuracy (95% CI)	0.541 (0.511-0.571)	0.612 (0.582-0.642)
Precision (95% CI)	0.579 (0.548-0.607)	0.576 (0.545-0.607)
Specificity (95% CI)	0.780 (0.754-0.806)	0.839 (0.816-0.861)
Sensitivity (95% CI)	0.302 (0.274-0.333)	0.850 (0.828-0.872)
*F*_1_-score (95% CI)	0.397 (0.367-0.427)	0.685 (0.656-0.714)

## Discussion

Although ChatGPT-4 Turbo demonstrated a slight ability to differentiate between pneumonia and nonpneumonia cases, this accuracy was overshadowed by the model’s strong bias, making its distinction between the two classes unreliable for clinical use. ChatGPT-4o is equally unreliable for clinical use.

Compared with Kermany et al [3], our ChatGPT results are subpar. ChatGPT’s best accuracy was 61.2% (ChatGPT-4o) in this study, compared to 92.8%. ChatGPT-4o’s sensitivity and specificity were also lower in this study: 85% and 38% compared to 93.2% and 90.1%, respectively. Noticeably, ChatGPT-4o’s specificity was very low comparatively. ChatGPT-4 Turbo’s sensitivity and specificity results were nearly reversed compared to its successor, indicating a substantial shift in predictive behavior. Our experiment only involved 1000 testing samples in total, while Kermany et al [3] trained with 5232 samples and tested another 624 samples.

Several challenges exist in using ChatGPT-4 Turbo for diagnosing pneumonia from chest x-ray radiographs. The model’s strong bias toward classifying images as nonpneumonia significantly affected the accuracy and other measures used to evaluate the model’s performance. The high number of false negatives could lead to delayed or missed diagnoses in a clinical setting.

A limitation of this study is that the lack of complex pattern recognition of pediatric pneumonia by ChatGPT may be anticipated as the program has likely not been fine-tuned to assess these types of patterns. However, numerous studies have mentioned that programs like ChatGPT may replace radiologists, but studies are needed to improve these programs, and radiologists will continue to be vital to health care [8]. By providing empirical evidence of the limitations of generalist AI models, this study underscores the need for task-specific fine-tuning and integration with computer vision models, which can help further develop these programs.

ChatGPT-4 has limitations when diagnosing pneumonia from chest x-ray radiographs as shown by this research. The model’s strong bias toward a nonpneumonia diagnosis, limited ability to distinguish between the two classes, and lack of specialized medical knowledge suggest that it may be unsuitable for clinical use currently. Further research and development are needed to address these limitations and explore the potential of integrating language models with other computer vision techniques to improve the accuracy and reliability of automated pneumonia diagnosis from chest x-rays.

## Abbreviations

AI: artificial intelligence
CNN: convolutional neural network
